# A Novel Group Cognitive Behavioral Therapy Approach to Adult Non-rapid Eye Movement Parasomnias

**DOI:** 10.3389/fpsyt.2021.679272

**Published:** 2021-07-01

**Authors:** David O'Regan, Alexander Nesbitt, Nazanin Biabani, Panagis Drakatos, Hugh Selsick, Guy D. Leschziner, Joerg Steier, Adam Birdseye, Iain Duncan, Seán Higgins, Veena Kumari, Paul R. Stokes, Allan H. Young, Ivana Rosenzweig

**Affiliations:** ^1^Sleep Disorder Centre, Nuffield House, Guy's Hospital, London, United Kingdom; ^2^Faculty of Life Sciences and Medicine, King's College London, London, United Kingdom; ^3^Department of Neurology, Guy's Hospital, London, United Kingdom; ^4^Department of Neuroimaging, Sleep and Brain Plasticity Centre, King's College London, Institute of Psychiatry, Psychology and Neuroscience, London, United Kingdom; ^5^Insomnia Clinic, Royal London Hospital for Integrated Medicine, London, United Kingdom; ^6^Basic and Clinical Neurosciences, King's College London, Institute of Psychiatry, Psychology and Neuroscience, London, United Kingdom; ^7^Centre for Cognitive Neuroscience, College of Health, Medicine and Life Sciences, Brunel University London, Uxbridge, United Kingdom; ^8^Department of Psychological Medicine, King's College London & South London and Maudsley NHS Foundation Trust, Institute of Psychiatry, Psychology and Neuroscience, Bethlem Royal Hospital, Beckenham, United Kingdom

**Keywords:** cognitive behavioral therapy, NREM parasomnia, parasomnia, treatment, therapy

## Abstract

**Background:** Following the success of Cognitive Behavioral Therapy (CBT) for insomnia, there has been a growing recognition that similar treatment approaches might be equally beneficial for other major sleep disorders, including non-rapid eye movement (NREM) parasomnias. We have developed a novel, group-based, CBT-program for NREM parasomnias (CBT-NREMP), with the primary aim of reducing NREM parasomnia severity with relatively few treatment sessions.

**Methods:** We investigated the effectiveness of CBT-NREMP in 46 retrospectively-identified patients, who completed five outpatient therapy sessions. The outcomes pre- and post- CBT-NREMP treatment on clinical measures of insomnia (Insomnia Severity Index), NREM parasomnias (Paris Arousal Disorders Severity Scale) and anxiety and depression (Hospital Anxiety and Depression Scale), were retrospectively collected and analyzed. In order to investigate the temporal stability of CBT-NREMP, we also assessed a subgroup of 8 patients during the 3 to 6 months follow-up period.

**Results:** CBT-NREMP led to a reduction in clinical measures of NREM parasomnia, insomnia, and anxiety and depression severities [pre- vs. post-CBT-NREMP scores: *P* (Insomnia Severity Index) = 0.000054; *P* (Paris Arousal Disorders Severity Scale) = 0.00032; *P* (Hospital Anxiety and Depression Scale) = 0.037]. Improvements in clinical measures of NREM parasomnia and insomnia severities were similarly recorded for a subgroup of eight patients at follow-up, demonstrating that patients continued to improve post CBT-NREMP.

**Conclusion:** Our findings suggest that group CBT-NREMP intervention is a safe, effective and promising treatment for NREM parasomnia, especially when precipitating and perpetuating factors are behaviorally and psychologically driven. Future randomized controlled trials are now required to robustly confirm these findings.

## Introduction

Non-Rapid Eye Movement (NREM) parasomnias, or arousal disorders, are common in adults, where they represent a constellation of different unwanted behaviors and experiences, arising from or associated with sleep, for example from sleep walking to sexsomnia (1). In addition to night-time symptoms, they can also result in next day excessive tiredness, as well as adversely affect mood, cognition, and quality of life (2). Genetic predisposition plays a role and it is most evident in sleepwalking (3). Arousal disorders can be an important cause of sleep-related injury (4, 5), and it is crucial that their severity can be reliably diagnosed and assessed. More recently, Arnulf et al. (5) developed the Paris Arousal Disorders Severity Scale (PADSS), which has been consistently demonstrated across different NREM parasomnia phenotypes to reliably monitor and measure the clinical symptoms and severity of arousal disorders.

The understanding of the exact neurobiology and the maladaptive arousal mechanisms that underlie phenotypes of NREM parasomnia remains in its infancy (3, 6–8). Management is commonly multifaceted with an emphasis on psychoeducation and ideally on non-pharmacological measures (3). Pharmacotherapy is nonetheless frequently used in the treatment of NREM parasomnias (9). However, it is not always effective or wanted by patients, often because of fear of side-effects and dependency (3). Treatment success rates vary between different NREM parasomnia phenotypes, and polypharmacy may be required (9). In some cases, certain treatments, such as antidepressants, can worsen or even precipitate parasomnia symptoms (10). As NREM parasomnias are often chronic conditions, pharmacological treatment may be required long-term, which is often undesirable, especially when the patient is a young adult. Even when pharmacotherapy is successful, NREM parasomnias can re-emerge following treatment cessation, particularly if priming and precipitating factors remain unaddressed (11).

Of note is that affective disorders, and especially anxiety disorder, may lead to an increased frequency of negative emotions in NREM parasomnia mentation, and that this in turn may further increase daytime anxiety (12). Moreover, it has been argued that the reported distress associated with parasomnia/nightmare experience may have a more significant impact on patients' quality of life, even more so than the frequency of parasomnic events [for an in-depth review of this topic please refer to (12)]. In keeping with this, to date, several psychotherapeutic approaches, for example: via Gestalt therapy (13) and imagery rehearsal therapy (14), have been shown to successfully target dysphoric parasomnias and to treat associated significant clinical distress.

In order to address the growing need for non-pharmacological therapies for NREM parasomnias (15), we have recently developed a novel, group-based, Cognitive Behavioral Therapy (CBT-NREMP) programme. The pathophysiological precipitants of NREM parasomnias suggest that CBT interventions, which address co-morbid insomnia, anxiety, stress and other relevant psychological difficulties, may be beneficial in its management (16). Our goal was therefore to primarily target factors which may trigger and maintain parasomnias over time, by incorporating and building-on core principles from the well-established and cost-effective model (17) Cognitive Behavioral Therapy for Insomnia (CBT-I) (18). The novel CBT for NREM parasomnia (CBT-NREMP; Supplementary Material) protocol includes a comprehensive programme that covers psychoeducation on the etiology of NREM parasomnias, sleep hygiene, sleep rescheduling to optimize homeostatic regulation, stimulus control to re-establish an association between the bed/bedroom and sleep, and specified body-based and cognitive relaxation techniques. By changing maladaptive sleep-related behaviors, thoughts and anxiety, CBT-NREMP treatment is specifically designed to target those priming and precipitating factors which cause parasomnias to persist over time. Moreover, it enables an individual to gain insight into their own thoughts as well as their emotional and behavioral processes regarding the self. The CBT programme is delivered in a safe group environment that additionally utilizes the spontaneity and creativity of the individual and the group. Here we report on the preliminary treatment outcomes of our novel CBT-NREMP programme.

## Materials and Methods

### Design, Ethics, and Data Collection

**Figure 1:**
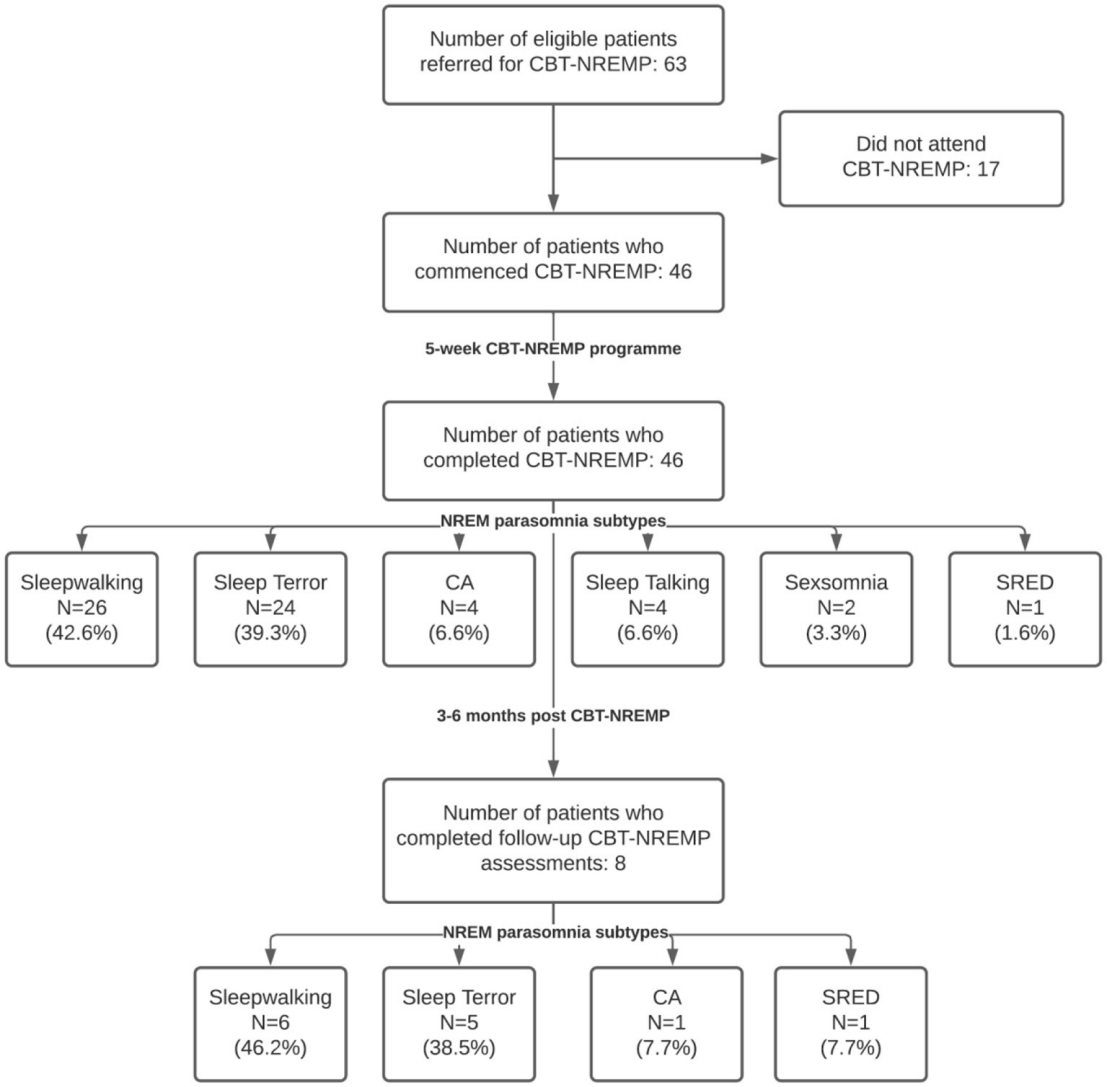
Flow diagram of the studied cohort. *Nota Bene:* some patients had *n* > 1 subtype of NREM parasomnia recorded. Percentages indicate the prevalence of each NREM parasomnia subtype in our cohort. CBT-NREMP, cognitive behavioral therapy for non-REM parasomnia; CA, confusional arousal; SRED, sleep-related eating disorder; NREM, non-REM; n, number.

All adult patients who had completed a whole programme (i.e., five sessions) of structured group CBT-NREMP between November 2018 and January 2020 were retrospectively identified, and their clinical findings, including demographics and the scores of several clinical questionnaires routinely used in our tertiary sleep disorder center, were collected from the center's clinical sleep database and analyzed. Altogether 46 patients were identified matching that criteria, and of those, a subgroup of eight patients were identified for whom 3 to 6 months follow up assessment findings were also available (Figure 1).

As per our clinical governance, framework the specified requirements to enroll in CBT-NREMP included a previously conducted video polysomnography (vPSG) investigation, and a confirmed diagnosis of NREM parasomnia by a qualified sleep physician, based on International Classification of Sleep Disorders third edition (ICSD-3) criteria (1). In addition to these inclusion criteria, all referred patients were screened by an experienced psychiatrist/psychologist, to confirm and assess their ability to participate in the group psychotherapy, as well as to ascertain the patient's ability to understand, speak and write English language, and to confirm their willingness and ability to give informed consent. The CBT-NREMP exclusion criteria included: co-morbid sleep disorders (apart from comorbid insomnia), current or past neurologic or psychiatric illness, traumatic brain injury, current alcohol and/or substance dependency disorders, developmental disorders and intellectual disability.

For the purposes of this study, the effectiveness of CBT-NREMP was evaluated by analyzing the outcomes of the three major clinical questionnaires from the clinical sleep database, including the Insomnia Severity Index (ISI) (19), Hospital Anxiety and Depression Scale (HADS) (20), and the Paris Arousal Disorders Severity Scale (PADSS) (5) at baseline, post-CBT-NREMP, and at follow-up (FU) 3 to 6 months later.

ISI is a self-rated scale, used to assess severity of insomnia in the clinical and research settings (21). The scale uses a seven-item self-report questionnaire that examines the nature, severity, and impact of insomnia. The evaluated dimensions include severity of sleep onset, sleep maintenance, and early morning awakening problems, sleep dissatisfaction, interference of sleep difficulties with daytime functioning, noticeability of sleep problems by others, and distress caused by the sleep difficulties. A five-point Likert scale is used to rate each item (e.g., 0 = no problem; 4 = very severe problem), yielding a total score ranging from 0 to 28 (21). Based on the total score the absence of insomnia (0–7); sub-threshold insomnia (8–14); moderate insomnia (15–21); or severe insomnia (22–28) can be identified (19). Similarly, HADS is also a self-rated scale, used to assess severity of depression and anxiety symptomatology (20). This 14-item scale includes seven items each for anxiety and depression subscales, where scoring for each item ranges from zero to three. A subscale score >8 denotes anxiety or depression. PADSS is a self-administered questionnaire designed to assess the severity of parasomnia (5). The scale has excellent psychometric properties, as well as valid and reliable subscales (22). It provides a means to assess the efficacy of new intervention treatments, as well as changes over longer periods of time. It consists of 17 items related to severity of parasomnia, with total score ranges from 0 to 50 (5); the scale has three parts, including an inventory of behaviors (PADSS-A), the frequency of episodes (PADSS-B), and the general consequences of the disorder (PADSS-C). The scale is self-completed and measured as follows: dangerous behaviors (17 items with three possible answers: never = 0, sometimes = 1, often = 2), frequency of episodes (equal to or more than two episodes per night = 6, one per night = 5, equal to or more than 1 episode per week = 4, equal to or more than 1 episode per month = 3, equal to or more than 1 episode per year = 2, <1 episode per year = 1, never had any = 0), and consequences of the disorder (5 items with three response options: never = 0, sometimes = 1, often = 2). The best cutoff score for the overall PADSS (range 0–50) was found at 13/14 and had high sensitivity (83.6%) and specificity (87.8%) (5). It has been shown in the past that the complexity of behaviors emerging from N3 sleep as assessed by the vPSG correlate positively with the scores for the PADSS-total, PADSS-A, and PADSS-C (5, 22).

The study was granted ethical approval by the Hospital Clinic Research Ethics Committee (Project-No-12025, GSTT NHS, UK) to retrospectively ascertain anonymized data in full compliance with the EU General Data Protection Regulation and the Declaration of Helsinki.

### CBT-NREMP Treatment

The structured group CBT programme consisted of five, weekly, 90 min CBT-NREMP sessions, with a maximum of eight participants per group. CBT-NREMP was conducted by an experienced sleep medicine psychiatrist or a trained psychologist according to a strict predetermined treatment protocol. Our protocol provided therapists with clear guidance on how to structure their therapy, as laid out in Supplementary Methods. The first sessions focused on building a therapeutic alliance and psychoeducation. The interventions sessions focused on both short- and long-term goals. Different cognitive and behavioral techniques (Supplementary Material) were applied to reach these goals. Homework was given in each session with the last session of therapy focusing on consolidation and relapse prevention. Experienced CBT-clinicians monitored adherence to the treatment principles in weekly group supervisions throughout the therapy period to ensure treatment fidelity. Clinical notes from the therapy sessions were regularly reviewed during supervisory sessions with focus on the initial phase of treatment, case formulation, treatment strategy and termination of therapy.

### Statistical Analyses

Descriptive statistics were used to summarize the data as mean ± standard deviation (SD), and with median, 25th and 75th percentiles for continuous non-parametric variables. Due to non-normality of the data, as assessed by Kolmogorov-Smirnov test, the non-parametric Wilcoxon signed rank test (paired comparisons) with Holm-Bonferroni corrections was used (5, 23) to test difference in severity between the CBT-NREMP group's insomnia (i.e., ISI), parasomnia (i.e., PADSS) and depressive and anxiety symptomatology (i.e., HADS) pre- and post-CBT scores. In addition, *post hoc analyses* were done for differences across the three time points, at the baseline, immediately following the CBT-NREMP and at the 3 to 6 months follow up (i.e., pre-, post-, and FU) for eight participants for whom follow-up data were available. A value of *P* < 0.05 was considered to be statistically significant and Holm-Bonferroni corrections were performed for the *post-hoc* analyses. The analyses were done using a statistical package R, version 4.0.2 for all statistical analyses (24).

## Results

Forty-six patients, of whom 25 were male (54.3%), aged 19 to 73 years-old (mean ± SD: 35.8 ± 11.4 years) underwent a structured, comprehensive 5 weeks CBT-NREMP group intervention. Patients were asked to complete baseline ISI, HADS, and PADSS assessments prior to starting CBT-NREMP, and the same assessments were subsequently completed after the CBT-NREMP intervention (Tables 1, 2).

**Table 1:** Outcomes of ISI, HADS, and PADSS assessments in 46 NREM parasomnia patients at baseline (Pre) and following the CBT-NREMP treatment (Post).

**Assessment**	**Pre**		**Post**	
	**Mean (SD)**	**Median (Q1, Q3)**	**Mean (SD)**	**Median (Q1, Q3)**
ISI	15.28 (4.36)	15 (12.25, 18)	12.09 (4.6)	12 (9.25, 15)
HADS	15.18 (6.55)	16 (11,19)	13.13 (5.98)	13 (8, 17.75)
HADS-A	8.14 (4.84)	7 (4.75, 12)	7.22 (4.24)	7 (4, 9.75)
HADS-D	7.02 (4.05)	6 (4,10)	5.91 (3.74)	6 (3,9)
PADSS	19.46 (6.32)	19 (16, 23.75)	17.53 (6.11)	17 (14,22)
PADSS-A	9.8 (4.67)	10 (6.25, 13.5)	8.41 (4.16)	8 (5,10)
PADSS-B	4.41 (1.11)	4 (4,5)	4.46 (1.21)	4 (4, 5.75)
PADSS-C	5.24 (1.78)	5 (4,7)	4.84 (2.01)	5 (3, 6.25)

*ISI, Insomnia Severity Index; HADS, Hospital Anxiety and Depression Scale (total score); HADS-A, Hospital Anxiety and Depression Scale-Anxiety subset score; HAD-D, Hospital Anxiety and Depression Scale—Depression subset score; PADSS, Paris Arousal Disorders Severity Scale (total score); PADSS-A, Paris Arousal Disorders Scale-subset A score; PADSS-B, Paris Arousal Disorders Scale subset-B score; PADSS-C, Paris Arousal Disorders Scale subset-C score. Q1, 25% percentile. Q3, 75% percentile. SD, standard deviation*.

**Table 2:** Results of Wilcoxon signed rank tests comparing pre- and post-CBT-NREMP intervention scores for ISI, HADS, and PADSS assessments in 46 NREM parasomnia patients.

**Assessment**	**Difference from Pre- to Post-CBT median (Q1, Q3)**	**Difference in median (95% CI)**	**Wilcoxon signed rank test**	***P*-value**
ISI	3 (0, 6.75)	3 (1, 6)	710.5	**0.000054**
HADS	1 (−1, 6)	3 (−0.84, 5.84)	514.5	**0.037**
HADS—A	1 (−1, 3)	0 (−1, 2.97)	512	0.089
HADS—D	1 (0, 2)	0 (−2, 3.5)	467.5	**0.034**
PADSS	1 (0, 3)	2 (−1.40, 6)	560	**0.00032**
PADSS—A	1 (−0.75, 2.75)	2 (0, 3.5)	600.5	**0.003**
PADSS—B	0 (0, 0)	0 (−1, 1)	71.5	0.826
PADSS—C	0 (−0.25, 1)	0 (−1, 2)	306.5	0.119

*ISI, Insomnia Severity Index; HADS, Hospital Anxiety and Depression Scale; PADSS, Paris Arousal Disorders Severity Scale. Q1, 25% percentile. Q3, 75% percentile. CI, confidence interval. CBT, cognitive behavioral therapy. Statistically significant values are shown in bold*.

At the baseline, patients' PADSS scores reflected the clinical severity of their untreated NREM parasomnia (mean PADSS score: 19.46 ± 6.32; Table 1). Patients scored moderately high on clinical measures of insomnia (ISI: 15.28 ± 4.36), with the baseline HADS outcome scores suggestive of subthreshold levels of anxiety and low mood (HADS-A: 8.14 ± 4.84 vs. HADS-D: 7.02 ± 4.05).

The CBT-NREMP intervention successfully reduced measures of clinical severity of NREM parasomnia (PADSS: *P*_Pre*vs*Post_ = 0.00032; Table 2). Further significant improvements were noted in clinical measures of insomnia (ISI_Pre*vs*Post_: *P* = 0.000054; Table 2), which were reduced to clinical subthreshold values (Table 1), as well as in patients' self-reported severity of anxiety and depressive symptoms (HADS_Pre*vs*Post_: *P* = 0.037; Table 2).

### Preliminary Findings on Sustainability of the CBT-NREMP Intervention

A subgroup of eight patients (17.4%) was followed after the CBT-NREMP intervention for up to 6 months (please also see Supplementary Material). By comparison to the socio-demographics of the larger group, the smaller subgroup consisted of younger (29.5 ± 8.1 years), predominantly female (six, 75%) patients, who at the outset reported higher clinical measures of severity of NREM parasomnia (PADSS scores: 24.75 ± 3.62; Supplementary Table 1) and anxiety (HADS-A: 11.25 ± 5.18; Supplementary Table 1).

Here, the CBT-NREMP intervention also significantly reduced the clinical measures of severity of NREM parasomnia and insomnia (Supplementary Table 2). These improvements were maintained, with further reduction in clinical measures of frequency and severity for NREM parasomnia and insomnia reported to continue for up to 6 months following the intervention (ISI: *P* = 0.042; PADSS: *P* = 0.041; Supplementary Table 2).

The CBT-NREMP intervention, however, did not lead to a statistically significant reduction in clinical measures of low mood and anxiety for this subgroup (HADS: *P* = 0.22). Nonetheless, the longitudinal reduction in the mean HADS scores was recorded across the assessment time-points (HADS *Pre*: 17.5 ± 8.64; *Post CBT-NREMP*: 14.88 ± 4.52; *FU* 3 *to* 6 months:11.88 ± 7.02; Supplementary Table 1), with the most consistent improvement reported to occur during the follow-up period of up to 6 months after the intervention (HADS-A: *P* = 0.057; Supplementary Table 2). This may suggest a delayed nature of this response, or its secondary development as a consequence of primary improvements in sleep measures.

## Discussion

The findings of our longitudinal study support the clinical utility for a novel CBT-NREMP intervention that targets distinct sleep, behavioral and emotional regulation factors. More specifically, we demonstrate that 5 weeks of a structured group CBT intervention in adult patients with NREM parasomnia can lead to a significant reduction in its severity. This is shown by a robust reduction in total PADSS and PADSS-A patients' scores (Table 1), both known to closely correlate with vPSG-ascertained severity (and complexity) of parasomnia behaviors that emerge from N3 sleep (5, 22).

In addition, we demonstrate that CBT-NREMP intervention can simultaneously lead to a clinically significant reduction in the patients' severity of insomnia, as evidenced by the reduction in the ISI scores. In our study, the ISI scores were robustly reduced from moderate to subthreshold values, with concomitant improvement in affective symptomatology (Table 1).

We also demonstrate that the effects of CBT-NREMP can be maintained, and that they continue to improve over a period of up to 6 months following the intervention (Supplementary Table 2). To the best of our knowledge, our study is the first to demonstrate the effectiveness, and arguably also the safety, of a structured CBT for adult NREM parasomnia.

Utilizing CBT in the treatment of sleep disorders holds substantial promise, and is clinically expanding (25). Where once medication-only treatments were favored, there has recently been a paradigm shift toward CBT-based interventions, which are viewed more favorably by patients (26), and treatment guidelines (27). CBT for insomnia (CBT-I) is already well-established as the gold-standard treatment, and principally operates by reducing perpetuating and precipitating factors associated with the condition (28). NREM parasomnias similarly manifest with priming (e.g., sleep loss, anxiety, stress, poor sleep hygiene), and precipitating factors (e.g., environmental noise) (16). Therefore, they should be amenable to a targeted CBT intervention, as our study amply demonstrates. Treating NREM parasomnias with CBT-NREMP, as opposed to medication, may have a number of potential advantages, including fewer known side-effects, and an explicit focus on treating the factors that may be responsible for perpetuating parasomnias in an effort to produce more durable effects.

Despite this, the body of literature on cognitive and behavioral interventions for NREM parasomnia is limited to case reports or smaller case-series, which often target just one parasomnia phenotype (29). In the past, selective application of CBT-I, mindfulness-based stress reduction and CBT for stress have been shown to helpfully target all phenotypes of NREM parasomnias (9). In our experience, patients with NREM parasomnia commonly struggle to benefit from other CBT paradigms, where they often feel apart from the rest of the group. For example, it can be understandably challenging for a patient with sleepwalking to engage in, and accept, a therapy which solely focuses on insomnia. Indeed, the development of our targeted group CBT-NREMP arose in part from this unmet patient need.

Despite the striking and sustainable improvements reported by our patients, several notable limitations merit further mention. Firstly, CBT-NREMP was designed as an economical and inclusive group intervention, which could be potentially delivered in a variety of clinical settings and that reliably targets diverse physiologic phenotypes of arousal disorders. Whilst this was beyond the scope of our study, future studies should ideally examine whether taking a stepped-care approach would be more beneficial for different settings or NREM parasomnia phenotypes, possibly avoiding any potential selection bias. For example, any such multifaceted CBT-NREMP intervention could arguably start with group therapy sessions that address common therapeutic targets in parasomnia (e.g., safety, sleep hygiene), with subsequent individual interventions focusing on specific and more complex phenotypes, such as trauma-related presentations and sexomnia.

Secondly, whilst the findings of our study suggest that a robust short term (e.g., 3 to 6 months) maintenance of CBT-NREMP effects is possible, this effect was only shown in eight, as opposed for 46 original study patients, due to unforeseen and early study closure during the Covid-19 pandemic. This smaller subgroup had a widely differing sociodemographic in that the patients were notably younger, they reported higher baseline anxiety, and they were predominantly women. Hence, the CBT-NREMP sustainability should be confirmed in a larger patient cohort, and the specific CBT-NREMP effects and their sustainability ideally recorded over a significantly longer period of time.

Another potential limitation worth mentioning is that our assessment was based primarily on patients' subjective reports. The self-reported scores, recorded in PADSS, ISI and HADS questionnaires are, however, widely used, and all three have been robustly validated for clinical and research purposes (5, 21, 30). Nonetheless, the subjective nature of patients' reports may arguably render any truly objective interpretation of CBT-NREMP's effectiveness invalid. We challenge the clinical significance of this limitation, given that the major aim of any clinical treatment of NREM parasomnia is primarily offered to ensure patients' safety, and secondly, to address the patients' symptoms according to their own criteria (9).

Taken together, the findings of our study demonstrate that structured group CBT for adult NREM parasomnia is a safe, effective, and a highly promising treatment. Due to its unique design, CBT-NREMP intervention may be especially effective in those patients in whom precipitating and perpetuating factors are likely behaviorally and psychologically driven. However, in order to reliably build on our preliminary study, future randomized controlled trials are required. Ideally, any such trial should include prospective multimodal physiologic and neuroimaging investigation to decipher neuromechanisms which underlie and promote differential effects of CBT-NREMP's intervention. Following this approach, it is hoped that with time we will also gain further insight into the role that patients' gender and their emotional fragility may play. Going forward, it would be important to understand how they may impact objective CBT-NREMP outcomes, including the electroencephalographic arousal signatures and their behavioral correlates.

## Data Availability Statement

The original contributions presented in the study are included in the article/Supplementary Material, further inquiries can be directed to the corresponding author/s.

## Ethics Statement

The studies involving human participants were reviewed and approved by the Hospital Clinic Research Ethics Committee (Project-No-12025, GSTT NHS, UK) to retrospectively ascertain anonymized data in full compliance with the EU General Data Protection Regulation and the Declaration of Helsinki. The patients/participants provided their written informed consent to participate in this study.

## Author Contributions

DO'R, AN, and IR: conceptualization. DO'R, AN, NB, PD, HS, GL, JS, AB, ID, SH, and IR: methodology and study administration. All authors contributed to drafting and reviewing the manuscript.

## Conflict of Interest

AY is employed by King's College London; Honorary Consultant SLaM (NHS UK). Paid lectures and advisory boards for the following companies with drugs used in affective and related disorders: Astrazenaca, Eli Lilly, Lundbeck, Sunovion, Servier, Livanova, Janssen, Allegan, Bionomics, Sumitomo Dainippon Pharma, COMPASS. Consultant to Johnson & Johnson and Livanova. Received honoraria for attending advisory boards and presenting talks at meetings organized by LivaNova. Principal Investigator in the Restore-Life VNS registry study funded by LivaNova. Principal Investigator on ESKETINTRD3004: An Open-label, Long-term, Safety and Efficacy Study of Intranasal Esketamine in Treatment-resistant Depression. Principal Investigator on The Effects of Psilocybin on Cognitive Function in Healthy Participants. Principal Investigator on The Safety and Efficacy of Psilocybin in Participants with Treatment-Resistant Depression (P-TRD). UK Chief Investigator for Novartis MDD study MIJ821A12201. Grant funding (past and present): NIMH (USA); CIHR (Canada); NARSAD (USA); Stanley Medical Research Institute (USA); MRC (UK); Wellcome Trust (UK); Royal College of Physicians (Edin); BMA (UK); UBC-VGH Foundation (Canada); WEDC (Canada); CCS Depression Research Fund (Canada); MSFHR (Canada); NIHR (UK). Janssen (UK). PRS reports grants and non-financial support from Corcept Therapeutics, a grant from H Lundbeck, non-financial support from Janssen Research and Development LLC, honoraria and non-financial support from Frontiers in Psychiatry, personal fees from Allergan, outside the submitted work. The remaining authors declare that the research was conducted in the absence of any commercial or financial relationships that could be construed as a potential conflict of interest.
