# Supplementary material for: A Novel Group Cognitive Behavioral Therapy Approach to Adult Non-rapid Eye Movement Parasomnias
Source: Front Psychiatry. 2021 Jul 1;12:679272. doi: 10.3389/fpsyt.2021.679272 (PMC8281294; doi:10.3389/fpsyt.2021.679272)
Supplement: Supplementary file 1 [file Data_Sheet_1.PDF]

Supplementary Material

1 Materials and Methods

CBT-NREMP protocol

The outpatient program was conducted by an experienced somnology therapist (e.g. psychiatrist or psychologist) and consisted of five sessions (90 minutes, weekly, over five weeks; group setting with eight patients). The course focused on psychoeducation about sleep, NREM parasomnia, cognitive skills, and relaxation training to manage stress and anxiety. The teaching was combined with daily homework assignments. The session outline was as follows:

Session 1 – Sleep and CBT-NREMP education

Psychoeducation about the nature and science of sleep, the impact of NREM parasomnias and explanation of the CBT-NREMP model was conducted. The programme covered a therapeutic rationale for treatment of NREM parasomnias with CBT, including the managing of the expectations. Further psychoeducation on NREM parasomnia etiology and an explanation of the Pressman model (Pressman 2007) was delivered, along with education on the stages of sleep (i.e. explanation of the hypnogram); daytime consequences of NREM parasomnias, and patients were taught how to assess how much sleep is truly required based on their own individual need. Further education on NREM parasomnia trajectories, their associated risks with appropriate reassurance was provided, along with essential advice on appropriate safety measures that each patient should undertake.

Moreover, during this core session, patients learned about the essentials of sleep hygiene and their habits/behaviors associated with sleep interference were explored. In addition, a clear instruction on ways of reducing/eliminating maladaptive sleep behaviour patterns was provided, including information on dietary factors (e.g. caffeine and alcohol intake), other lifestyle factors (e.g. nicotine use before bed, lack of a bedtime routine) and bedroom factors (e.g. noise, temperature, clock watching). Finally, patients were introduced and instructed on how to use sleep diaries.

Session 2 – Stimulus control, anchoring the day, and sleep rescheduling

During the second core session, patients were taught about stimulus control. Stimulus control helps patients to reduce the association of the bedroom as being a place of wakeful, purposeful activity. It limits bed and bedroom activities to sleep, sex and getting dressed only. It further emphasises the importance of only going to bed when sleepy. The 15-minute rule encourages individuals to leave the bedroom and to engage in a relaxing activity, if they have not slept after 15-minutes (defined as a time period they guess, rather than calculate via an alarm or via clock watching). Individuals return to bed when they feel sleepy again. This rule is maintained throughout the night and further weakens the association between the bed and wakefulness.

Anchoring the day, teaches the importance of rising at (or before) a set time each morning, seven days a week, regardless of how well an individual has slept the previous night. This anchors the circadian rhythm and ensures that the accumulation of sleepiness starts at the same time every day, which makes the onset of sleep at night more predictable. The accumulation of sleepiness is further reinforced by avoidance of napping (intentional or unintentional) during the day.

In sleep rescheduling, the initial requirement is to limit the time in bed to no more than the average estimated time spent asleep each night (calculated from each individual's sleep diary). By subtracting their average sleep time from their rising (anchor) time, a threshold time is obtained. Participants were advised that they can go to bed when they reach that threshold time and they are sleepy. As the sleep efficiency improves, the time spent in bed can be increased.

Session 3 - Sleep diary review, buffer zone, progressive muscle relaxation, and managing anxiety at night

During the third core session, patients were further taught and encouraged to include a buffer zone (usually lasting 1.5-2 hours) before their sleep threshold. During this buffer zone, a pre-bed routine was advised (e.g. commencing with a warm bath), and "putting the day to bed" by dealing constructively with worries or anxieties arising from the day e.g. via use of a reflective diary of what has gone well and not so well during the day. The buffer zone is used solely for relaxation, and as a "wind down" period, during which all work-related and stimulating activities are stopped.

Progressive Muscular Relaxation (Perlis et al. 2005) was introduced, and supported by an audio guide, which was given to participants.

Participants are also taught how to cope with unwanted thoughts or worries that arise during the night e.g. via use of a thought algorithm and via thought stopping techniques, as well as guided imagery.

Session 4 - Mindfulness-based body scan intervention

Participants were encouraged to bring in their own pillows and blankets for this session. Yoga mats, blankets and additional pillows were also provided. Following an introduction to the session, participants were guided through a lying down, 45-minute, mindfulness-based body scan intervention. They were encouraged to practice this at home during the day, aided by recommended supporting resources.

Session 5 - Bringing it all together, and forward planning

The sessions were reviewed, aided by drawing a map of a typical day, indicating where each technique and strategy can be used. Participants were reminded how to use their sleep efficiencies to alter their sleep threshold times. Time was provided for individual queries, and guidance was given on realistic expectations over the coming weeks, with encouragement to continue practicing and implementing the techniques.

2 Additional Supplementary Tables

The preliminary analyses for the subgroup of eight patients who were additionally assessed following three to six months after the accomplished CBT-NREMP protocol are presented below.

Table S1. Outcome scores for ISI, HADS and PADSS assessments in a subgroup of 8 patients.

Assessment	Pre		Post		F/U	
	Mean (SD)	Median (Q1, Q3)	Mean (SD)	Median (Q1, Q3)	Mean (SD)	Median (Q1, Q3)
ISI	15.38 (2.72)	15 (14.5, 16.75)	12.38 (2.88)	12 (11.75, 14)	8.75 (3.49)	9 (6.5, 10.25)
HADS	17.5 (8.64)	17.5 (13, 21.5)	14.88 (4.52)	13 (12, 15.75)	11.88 (7.02)	9.5 (6.75, 15.25)
HADS_A	11.25 (5.18)	11.5 (8, 14.5)	10 (3.12)	8.5 (7.75, 13.25)	7.5 (4.07)	6 (5.5, 9)
HADS_D	6.25 (3.62)	6 (3.5, 8.5)	4.88 (2.85)	4.5 (3.75, 6.5)	4.38 (3.42)	2.5 (2, 7.25)
PADSS	24.75 (3.62)	24 (22.75, 26)	12.38 (2.88)	12 (11.75, 14)	13.38 (3.62)	14 (9.75, 17)
PADSS_A	13.88 (3.23)	13.5 (11, 15.25)	9.25 (3.2)	10 (8, 10)	5.62 (1.69)	6.5 (4, 7)
PADSS_B	4.75 (1.16)	4.5 (4, 6)	4.25 (1.04)	4 (3.75, 5)	3.38 (0.52)	3 (3, 4)
PADSS_C	6.12 (0.64)	6 (6, 6.25)	5.88 (1.64)	6.5 (4, 7)	4.38 (1.85)	4 (3, 6)

Abbreviations: ISI, Insomnia Severity Index; HADS, Hospital Anxiety and Depression Scale (total score); HADS-A, Hospital Anxiety and Depression Scale-Anxiety subset score; HAD-D, Hospital Anxiety and Depression Scale - Depression subset score; PADSS, Paris Arousal Disorders Severity Scale (total score); PADSS-A, Paris Arousal Disorders Severity Scale-subset A score; PADSS-B; Paris Arousal Disorders Severity Scale subset-B score; PADSS-C, Paris Arousal Disorders Severity Scale subset-C score. Q1, 25% percentile. Q3, 75% percentile. SD, standard deviation.

Table S2. Post hoc pairwise group comparison through Wilcoxon signed rank tests comparing pre-, post-and FU-CBT invention scores for ISI, HADS and PADSS assessments in a subgroup of 8 patients.

		Wilcoxon signed rank test statistics	*P*-value	Holm-Bonferroni adjusted *P*-value
**ISI**				
Pre	Post	31.5	0.065	0.065
Pre	F/U	36	**0.014**	**0.042**
Post	F/U	35	**0.021**	**0.042**

**PADSS**				
Pre	Post	36	**0.014**	**0.041**
Pre	FU	36	**0.014**	**0.041**
Post	FU	36	**0.014**	**0.041**
**PADSS-A**				
Pre	Post	36	**0.013**	**0.04**
Pre	FU	36	**0.014**	**0.04**
Post	FU	36	**0.014**	**0.04**
**PADSS-B**				
Pre	Post	3	0.371	0.371
Pre	FU	15	0.057	0.17
Post	FU	10	0.089	0.178
**PADSS-C**				
Pre	Post	13.5	0.589	0.589
Pre	FU	25.5	0.061	0.121
Post	FU	28	**0.02**	0.06
**HADS**				
Pre	Post	25.5	0.326	0.326

Pre	FU	33	**0.042**	0.125
Post	FU	31	0.078	0.156
**HADS-A**				
Pre	Post	24	0.44	0.44
Pre	FU	32.5	**0.049**	0.147
Post	FU	32	0.057	0.147
**HADS-D**				
Pre	Post	10.5	0.498	0.996
Pre	FU	16	0.292	0.876
Post	FU	21	0.719	0.996

Abbreviations: ISI, Insomnia Severity Index; PADSS, Paris Arousal Disorders Severity Scale (total score); PADSS-A, Paris Arousal Disorders Severity Scale-subset A score; PADSS-B; Paris Arousal Disorders Severity Scale subset-B score; PADSS-C, Paris Arousal Disorders Severity Scale subset-C score; HADS, Hospital Anxiety and Depression Scale (total score); HADS-A, Hospital Anxiety and Depression Scale-Anxiety subset score; HAD-D, Hospital Anxiety and Depression Scale - Depression subset score. FU, follow-up. *Statistically significant values (both corrected and uncorrected) are shown in bold.*
